# Biomechanical Behavior of Polyether Ether Ketone Composite Dental Implants: A Three-Dimensional Finite Element Analysis

**DOI:** 10.7759/cureus.90953

**Published:** 2025-08-25

**Authors:** Meenakshi Thimmappa, Greeshma BJ, Neeraja B, Ruchira Paul, Prathyusha Movva, Krishnaprasad TR

**Affiliations:** 1 Prosthodontics and Crown and Bridge, The Oxford Dental College, Bengaluru, IND; 2 Prosthodontics and Crown and Bridge, Pacific Dental College and Hospital, Udaipur, IND

**Keywords:** 3d finite element analysis, marginal bone loss, peek composite, polyether ether ketone (peek), stress shielding

## Abstract

Aims: This study aimed to assess the biomechanical performance of polyether ether ketone implants in their unmodified form and composite forms reinforced with carbon fibers, glass fibers, hydroxyapatite, and strontium-hydroxyapatite, using finite element analysis across both low- and high-density bone conditions.

Materials and methods: By using a three-dimensional computer-aided design software (SolidWorks, SolidWorks Corp., Waltham, Massachusetts, United States), finite element models of both unmodified and composite polyether ether ketone implants were developed for low and high bone densities. These models were analyzed using the ANSYS 8.0 simulation platform (Ansys, Inc., Canonsburg, Pennsylvania, United States) under vertical, oblique, and combined loading conditions, applying a force of 100 newtons. Stress and deformation levels were assessed using the von Mises stress criteria.

Results: The unmodified polyether ether ketone implant showed the highest stress and deformation, whereas the carbon fiber-reinforced implant showed the lowest. Stress was more pronounced in low-density bone. All implants concentrated stress in the cervical region. For the unmodified implant, stress values were 34.59, 48.8, and 82.9 megapascals under vertical, oblique, and combined loads, respectively. In comparison, the carbon fiber-reinforced implant showed values of 28.53, 44.88, and 70.95 megapascals under the same conditions.

Conclusion: The carbon fiber-reinforced polyether ether ketone implant demonstrated the most favorable biomechanical characteristics, suggesting its potential for effective clinical use, especially across varying bone densities.

## Introduction

Titanium dental implants exhibit high survival rates, ranging from 90% to 96.5%, attributed to their favorable physical, mechanical, and biological properties [1]. However, implant failure can still occur, primarily due to the loss of osseointegration. One of the contributing factors to this is the stress shielding effect [2], a biomechanical phenomenon where reduced bone stimulation leads to bone resorption. This occurs because titanium's elastic modulus is 110 gigapascal (GPa) [3], which significantly exceeds that of both cortical bone (14 GPa) and cancellous bone (1.37 GPa) [4], creating a mechanical mismatch that limits natural stress transfer to the surrounding bone.

In addition to stress shielding, titanium implants have several clinical limitations, including the potential for hypersensitivity reactions [5], poor aesthetics in thin gingival biotypes [6], and radiologic limitations, such as artifacts in magnetic resonance imaging scans [7]. To address these concerns, alternative biomaterials have been explored. One such promising candidate is polyether ether ketone (PEEK), a member of the polyaryl ether ketone (PAEK) family. PEEK offers multiple advantages over traditional metal and ceramic implants: it is lightweight, easily machinable into complex geometries, cost-effective, and available in a range of mechanical properties.

However, unmodified PEEK exhibits limited osseointegration. To enhance its biological performance, modifications such as surface treatments and bioactive reinforcement have been introduced [8,9]. These approaches have led to the development of conventional PEEK composites, including carbon fiber-reinforced (CFR-PEEK), glass fiber-reinforced (GFR-PEEK), hydroxyapatite-reinforced (HA-PEEK), and strontium-hydroxyapatite-reinforced (Sr-HA-PEEK) variants [10].

While previous studies have evaluated the performance of unmodified PEEK and compared it with titanium implants [11], the biomechanical behavior of reinforced PEEK composites remains underexplored. Therefore, the present study was designed to perform a three-dimensional (3D) finite element analysis (FEA) to assess and compare the biomechanical efficiency of unmodified and composite PEEK implants, considering both low- and high-density bone conditions.

The primary objective was to analyze occlusal load distribution, associated marginal bone loss, and the stress shielding effect. The secondary objective was to identify the most biomechanically favorable PEEK composite implant for dental applications.

A null hypothesis was proposed: there would be no significant difference in biomechanical performance, specifically in stress distribution, marginal bone loss, and stress shielding, between unmodified and composite PEEK implants.

## Materials and methods

Aim, design, and setting of the study

Using 3D computer-aided design by SolidWorks (SolidWorks Corp., Waltham, Massachusetts, United States), finite element models of unmodified PEEK and four modified composite PEEK (CFR-PEEK, GFR-PEEK, HA-PEEK, and Sr-HA-PEEK) implants were fabricated for low and high bone densities. Stress analysis was assessed under 100 N axial, 30° oblique, and combined axial and non-axial forces concerning two different bone qualities. Hence, a total of 30 finite element models concerning the mandibular first molar region were constructed. FEA was performed using the ANSYS version 8.0 preprocessor software (Ansys, Inc., Canonsburg, Pennsylvania, United States). The study was conducted at Pacific Dental College and Hospital in Udaipur, India, and was reviewed and approved by the institute's Institutional Research Review Board (IRRB) (approval number: PDCH/18/EC-124).

Material properties considered: model construction and material properties

Material properties were adopted from established literature sources [4,6,9,12-17] and are presented in Table 1. For consistency, all 30 models maintained uniform dimensions across the implant, abutment, prosthesis, luting cement, and bone model. These parameters were consistent with the dimensions reported by Sarot et al. [16].

**Table 1:** Young's modulus and Poisson's ratio of materials PEEK: polyether ether ketone; CFR-PEEK: carbon fiber-reinforced polyether ether ketone; GFR-PEEK: glass fiber-reinforced polyether ether ketone; HA-PEEK: hydroxyapatite-reinforced polyether ether ketone; Sr-HA-PEEK: strontium-hydroxyapatite-reinforced polyether ether ketone

Types	Study	Young's modulus	Poisson's ratio
Unmodified PEEK	Bathala et al. [6]	3-4 GPa	0.38
CFR-PEEK	Sarot et al. [16]	18 GPa	0.39
GFR-PEEK	Rahmitasari et al. [12]	12 GPa	0.4
HA-PEEK	Song et al. [9]	5-7 GPa	0.3
Sr-HA-PEEK	Wong et al. [15]	10.7 GPa	0.4
Cortical bone	Kitagawa et al. [4]	14 GPa	0.30
Cancellous bone	Chang et al. [14]	1.37 GPa for high density and 0.8 GPa for low density	0.30 (for both bone densities)
Zinc phosphate cement	Sarot et al. [16]	22.4 GPa	0.25
Polytetrafluoroethylene	Sattar et al. [17]	0.575 GPa	0.46
Feldspathic porcelain	Sarot et al. [16]	69 GPa	0.3

The implant system consisted of a cylindrical, threaded-type implant with an external hex connection, measuring 11.5 mm in length and 4.2 mm in diameter. The abutment was made from unmodified PEEK with a screw-retained design, measuring 6 mm in length and 4 mm in diameter at the implant interface. The access cavity was sealed using polytetrafluoroethylene (Teflon) tape with a thickness of 0.1 mm. Zinc phosphate cement, with a film thickness of 0.1 mm, was used for luting. The prosthesis was a feldspathic porcelain-fused-to-cobalt-chromium alloy crown, designed for the mandibular first molar, with a height of 7 mm, an occlusal width of 11 mm, and a cervical width of 9 mm. The bone model included representations of both low- and high-density bone; the low-density model comprised cancellous bone surrounded by a 1 mm cortical layer, while the high-density model featured a 2 mm cortical layer [18].

**Figure 1:**
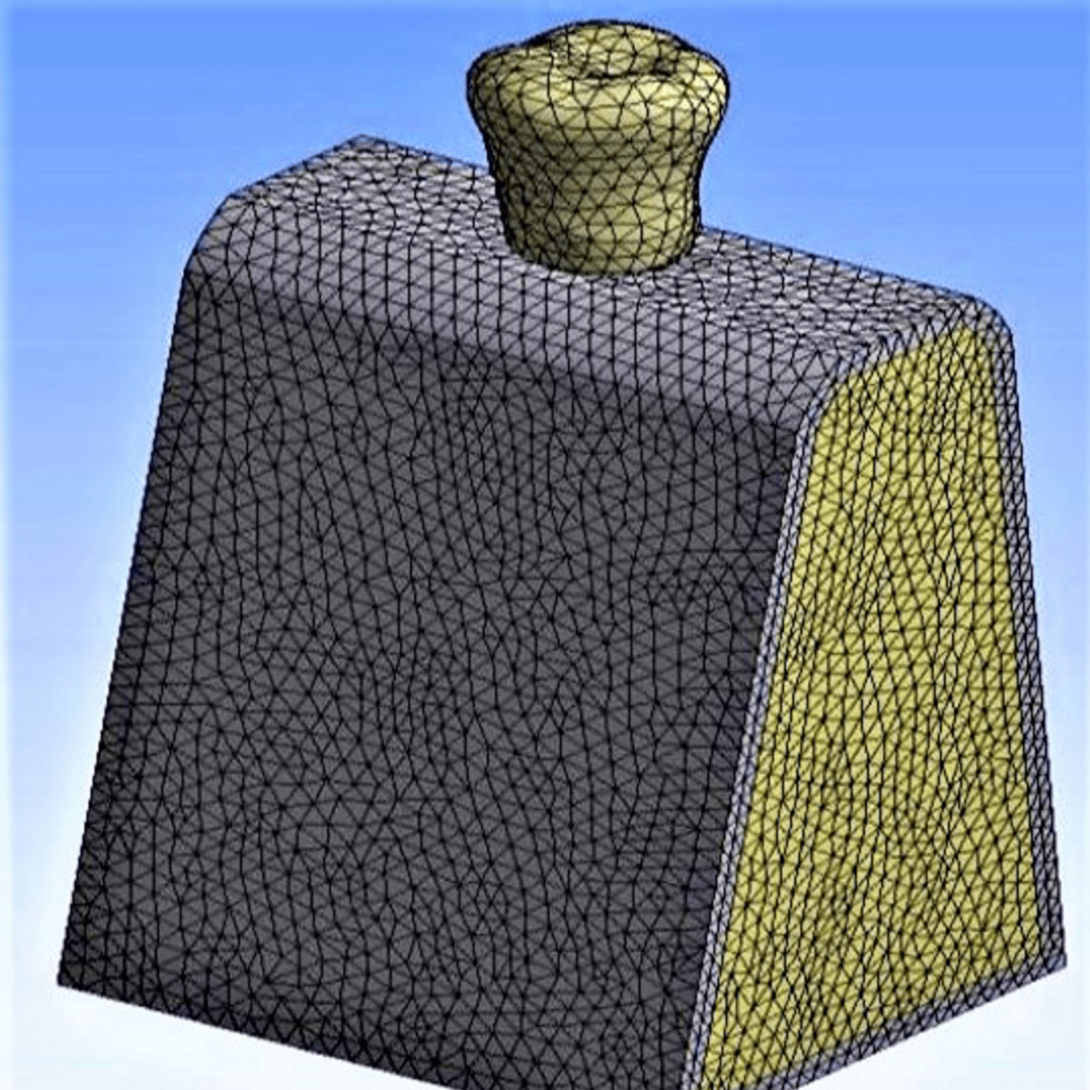
Finite element model meshwork

A SOLID92 10-node tetrahedral element was used for meshing the 3D model (Figure 1). The total number of elements and nodes for each model is summarized in Table 2.

**Table 2:** Number of elements and nodes

Components	Elements	Nodes
Crown	1981	3266
Abutment	14894	23852
Implant	31862	48132
Cortex	28256	45042
Cancellous	42237	61165
Cement	315	2264
Teflon	211	1555
Total	119756	185276

Boundary conditions

**Figure 2:**
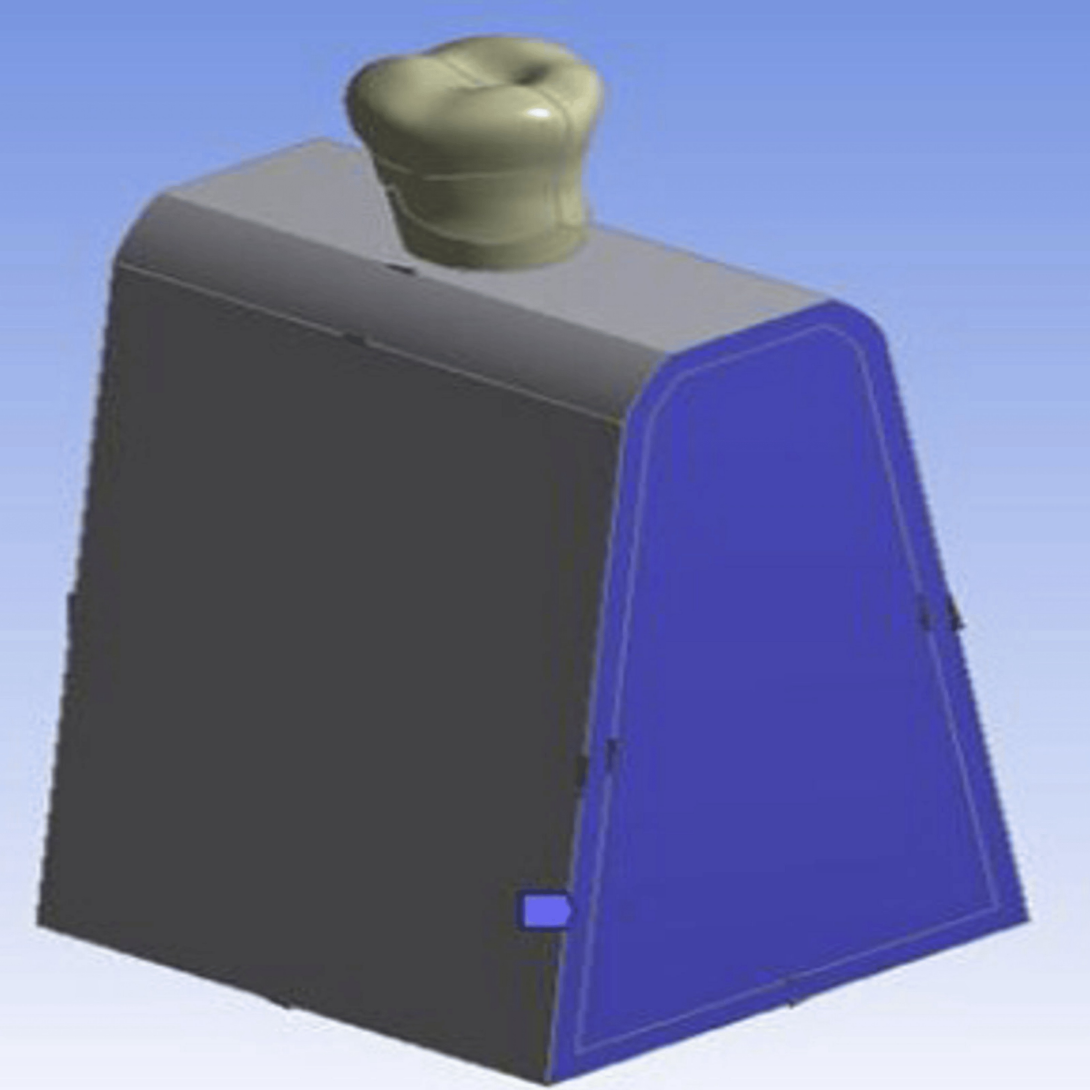
Boundary conditions considered fixed and frictionless

The boundary conditions were considered fixed, and there was no friction present between the components of the model. Hence, the implant was assumed to be 100% osseointegrated, and also the materials were assumed to be homogenous and isotropic (Figure 2).

Validation of the finite element model

**Figure 3:**
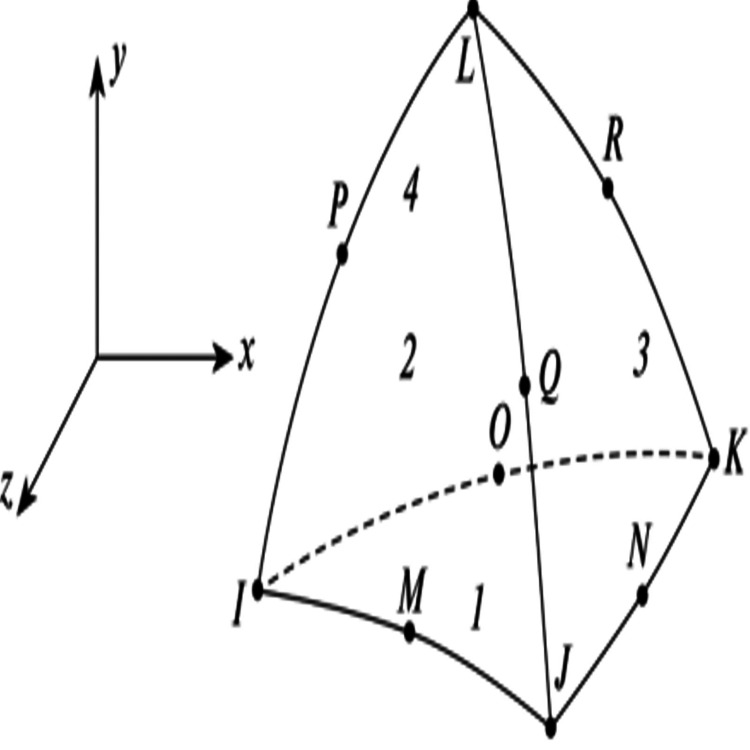
SOLID92 3D 10-node tetrahedral structural solid

A convergence study was conducted by meshing the model using tetrahedral elements, with each node possessing three degrees of freedom. The SOLID92 element, known for its quadratic displacement behavior, was selected due to its suitability for modeling complex and irregular meshes. This element is defined by 10 nodes (I, J, K, L, M, N, O, P, Q, R) [19], with each node allowing translations along the x-, y-, and z-axes (Figure 3). Additionally, this element type supports features such as plasticity, creep, swelling, stress stiffening, large deflection, and large strain. Mesh quality was evaluated using skewness, a key metric that assesses how close a mesh cell is to the ideal shape. Validation showed that most elements had skewness values between 0 and 0.25, indicating high mesh quality (Table 3). According to the skewness metric spectrum, values near 0 represent equilateral cells (excellent), while values approaching 1 indicate degenerated cells, typically with nearly coplanar nodes in 3D or colinear nodes in 2D [19]. Based on these criteria, all 30 finite element models were confirmed to be suitable and were utilized for linear static analysis using the ANSYS version 8.0 preprocessor software.

**Table 3:** Skewness values and cell quality

Value of skewness	Cell quality
1	Degenerate
0.9-<1	Bad
0.75-0.9	Poor
0.5-0.75	Fair
0.25-0.5	Good
>0-0.25	Excellent
0	Equilateral

Loading conditions

In this study, three static loading conditions were applied to the occlusal surface of the mandibular first molar prosthesis, namely, a 100 N vertical force along the long axis of the implant (axial loading), a 100 N force at a 30-degree angle to the long axis (oblique loading), and a combination of both vertical and oblique forces (combined loading), to simulate functional and parafunctional masticatory loads. These loads were evaluated under two bone density models, that is, low density (1 mm cortical layer over the cancellous bone) and high density (2 mm cortical layer over the cancellous bone), to assess stress distribution and deformation using linear static FEA.

The von Mises stress was analyzed using a color gradient ranging from blue (lowest stress) to red (highest stress). The resultant force was measured in megapascal (MPa).

## Results

In this study, the maximum von Mises stress criterion was employed, as it is well-suited for evaluating the mechanical response of rigid materials under load. A force of 100 N was applied in three configurations: vertically, at a 30° oblique angle, and as a combination of both. Table 4 presents the resulting von Mises stress values and deformation for both low- and high-density bone models.

**Table 4:** von Mises stress and deformation in LDB and HDB PEEK: polyether ether ketone; CFR-PEEK: carbon fiber-reinforced polyether ether ketone; GFR-PEEK: glass fiber-reinforced polyether ether ketone; HA-PEEK: hydroxyapatite-reinforced polyether ether ketone; Sr-HA-PEEK: strontium-hydroxyapatite-reinforced polyether ether ketone; LDB: low-density bone; HDB: high-density bone

Types of PEEK implants	Vertical			30° oblique		Combined	
	Maximum von Mises stress (MPa)		Maximum deformation (mm)	Maximum von Mises stress (MPa)	Maximum deformation (mm)	Maximum von Mises stress (MPa)	Maximum deformation (mm)
Unmodified PEEK	LDB	34.59	0.01	48.8	0.03	82.90	0.37
	HDB	20.7	0.006	49.3	0.03	70.02	0.033
CFR-PEEK	LDB	28.53	0.01	44.88	0.03	70.95	0.02
	HDB	17.5	0.005	44.56	0.02	62.09	0.033
GFR-PEEK	LDB	29.79	0.01	45.69	0.03	73.58	0.02
	HDB	18.27	0.005	45.73	0.03	63.72	0.03
HA-PEEK	LDB	32.84	0.01	47.53	0.03	79.62	0.02
	HDB	19.92	0.006	48.19	0.03	68.78	0.032
Sr-HA-PEEK	LDB	30.15	0.01	45.9	0.03	74.38	0.02
	HDB	18.53	0.006	46.13	0.03	64.65	0.032

Among the materials tested, unmodified PEEK exhibited the highest von Mises stress and deformation, while CFR-PEEK demonstrated the lowest values under all loading conditions. This was consistent across both low- and high-density bone scenarios. Additionally, stress concentrations were higher in low-density bone compared to high-density bone. Notably, lower von Mises stress values were observed under vertical loading compared to oblique and combined loading conditions.

## Discussion

FEA commonly uses two failure criteria: maximum principal stress for brittle materials and von Mises stress for ductile or rigid materials. The von Mises criterion, based on the distortion energy theory, considers all six components of stress in 3D and is widely used to evaluate yielding in rigid materials [20].

While extensive research has explored unmodified PEEK [3,6,21-24], fewer studies have examined the biomechanics of composite PEEK implants under functional loading. This study investigated the stress response of unmodified and modified PEEK implants (CFR-PEEK, GFR-PEEK, HA-PEEK, Sr-HA-PEEK) under 100 N axial, oblique, and combined forces in low- and high-density bone using FEA. The key observations indicate that unmodified PEEK consistently demonstrated the highest von Mises stress and deformation, whereas CFR-PEEK exhibited the lowest values across all bone densities and load types. Additionally, low-density bone showed significantly higher stress concentrations compared to high-density bone, and vertical loading generated lower stress levels than oblique or combined loading.

**Figure 4:**
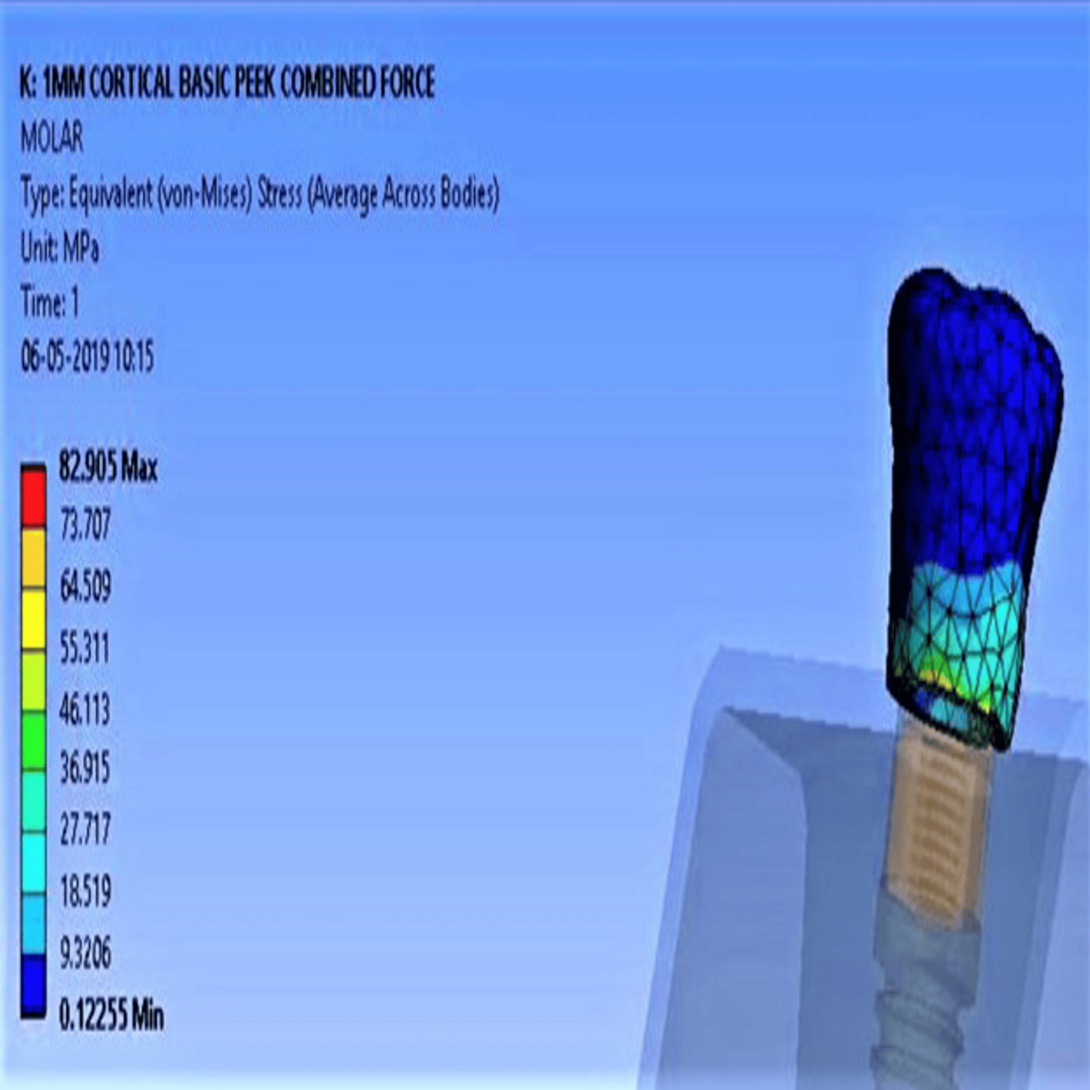
Maximum stress concentration for unmodified PEEK with low-density bone PEEK: polyether ether ketone

**Figure 5:**
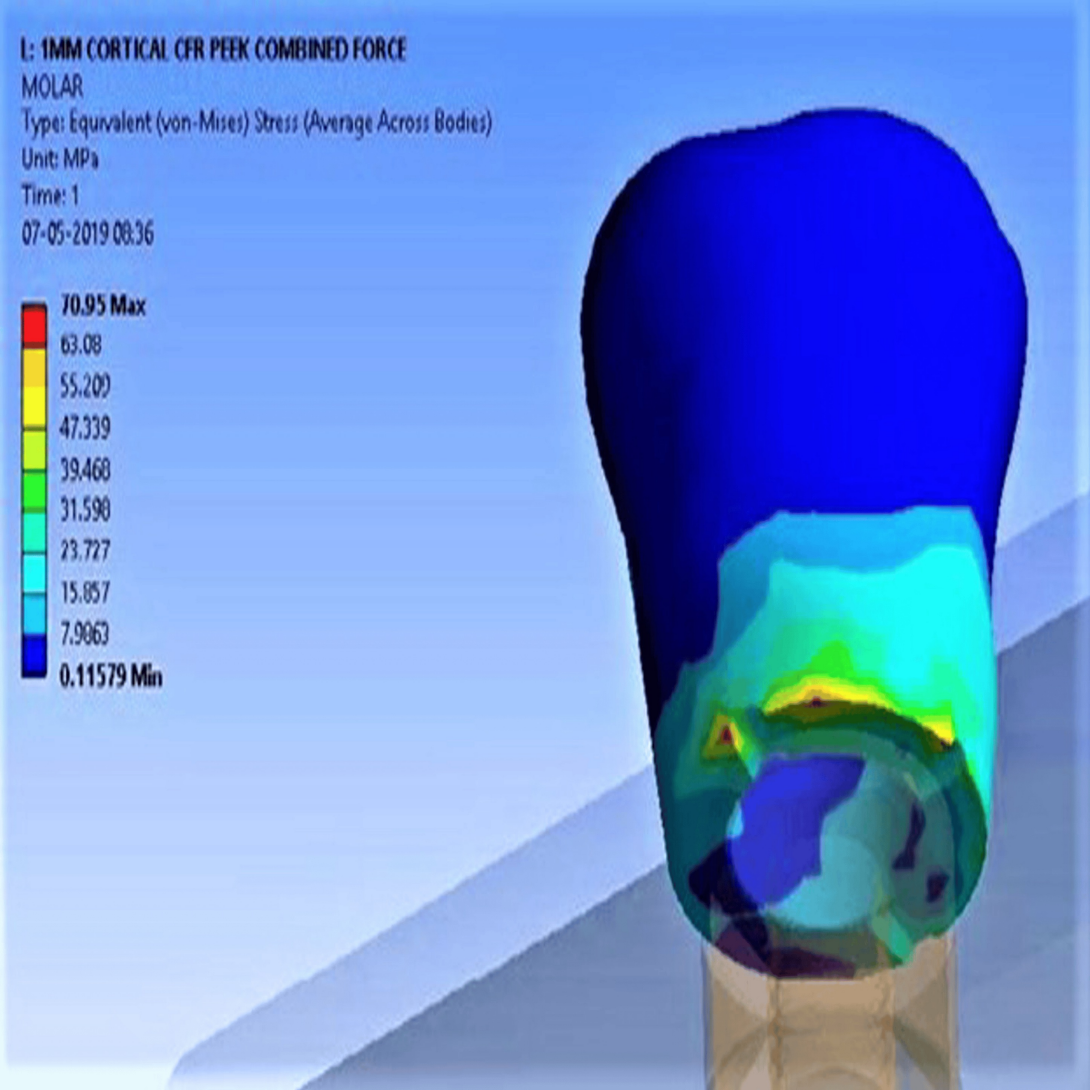
Minimum stress concentration for CFR-PEEK with low-density bone CFR-PEEK: carbon fiber-reinforced polyether ether ketone

Under low-density bone, unmodified PEEK displayed stress values of 34.59 MPa (vertical), 48.8 MPa (oblique), and 82.9 MPa (combined Figure 4). CFR-PEEK had significantly lower values: 28.53 MPa, 44.88 MPa, and 70.95 MPa (Figure 5), respectively. Deformation values for unmodified PEEK were 0.01 mm (axial), 0.03 mm (oblique), and 0.037 mm (combined). Composite PEEKs showed reduced deformation across all load conditions.

**Figure 6:**
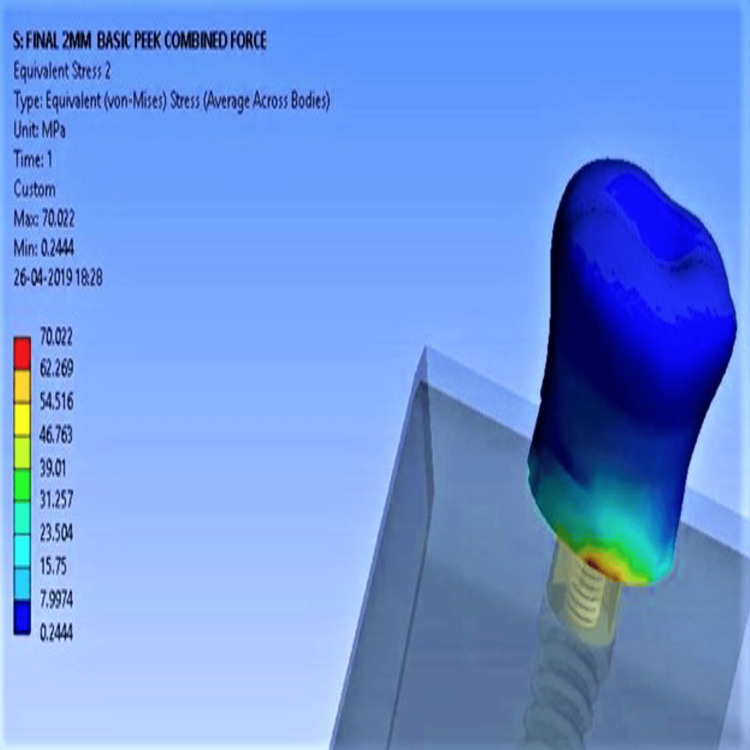
Maximum stress concentration for unmodified PEEK with high-density bone PEEK: polyether ether ketone

**Figure 7:**
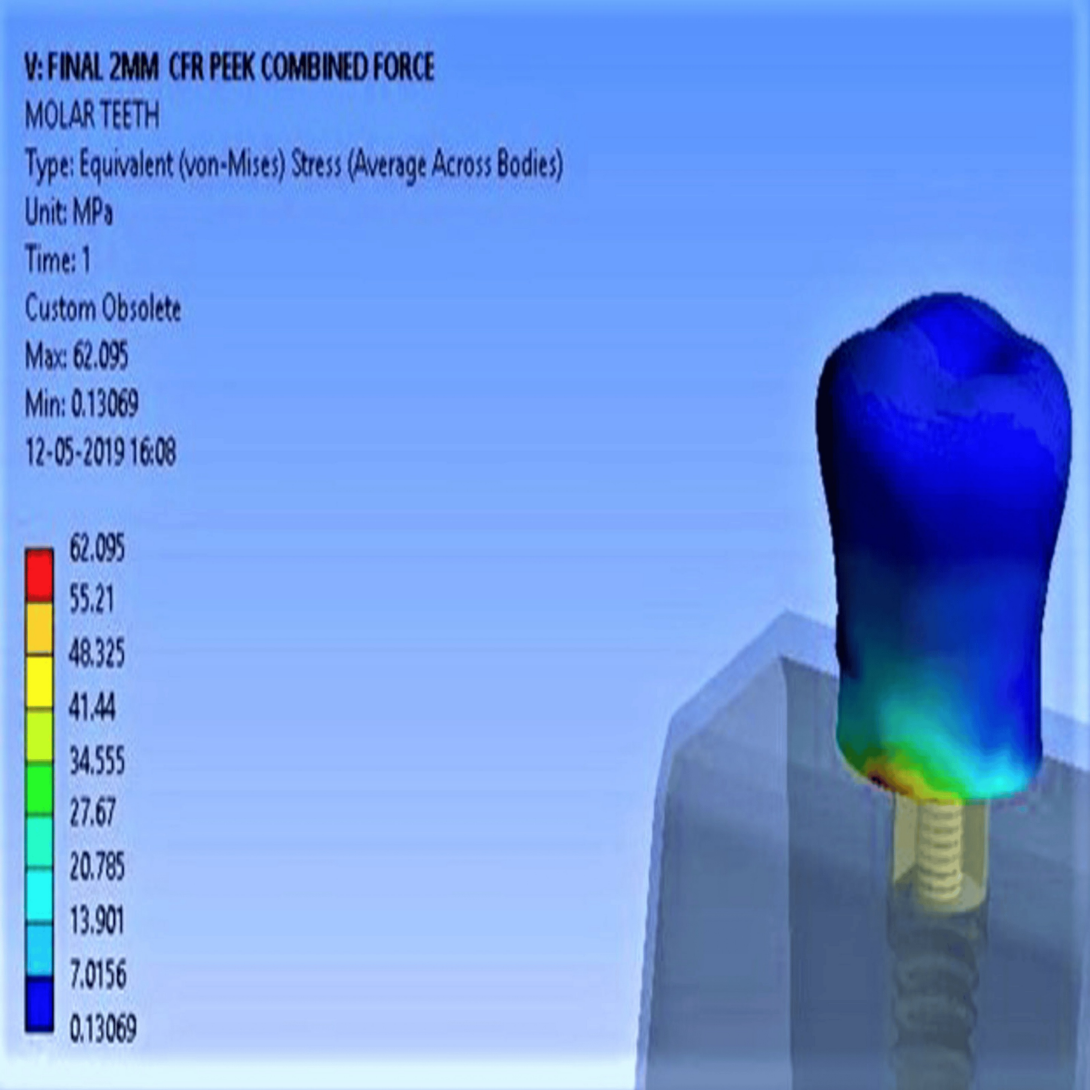
Minimum stress concentration for CFR-PEEK composite with high-density bone CFR-PEEK: carbon fiber-reinforced polyether ether ketone

With high-density bone, stress values were lower overall. Unmodified PEEK recorded 20.7 MPa (vertical), 49.3 MPa (oblique), and 70.02 MPa (combined Figure 6), while CFR-PEEK showed the lowest: 17.5 MPa (vertical), 45.56 MPa (oblique), and 62.09 MPa (combined Figure 7). Deformation differences under combined forces were minimal across all implants, with values ranging from 0.03 to 0.033 mm.

**Figure 8:**
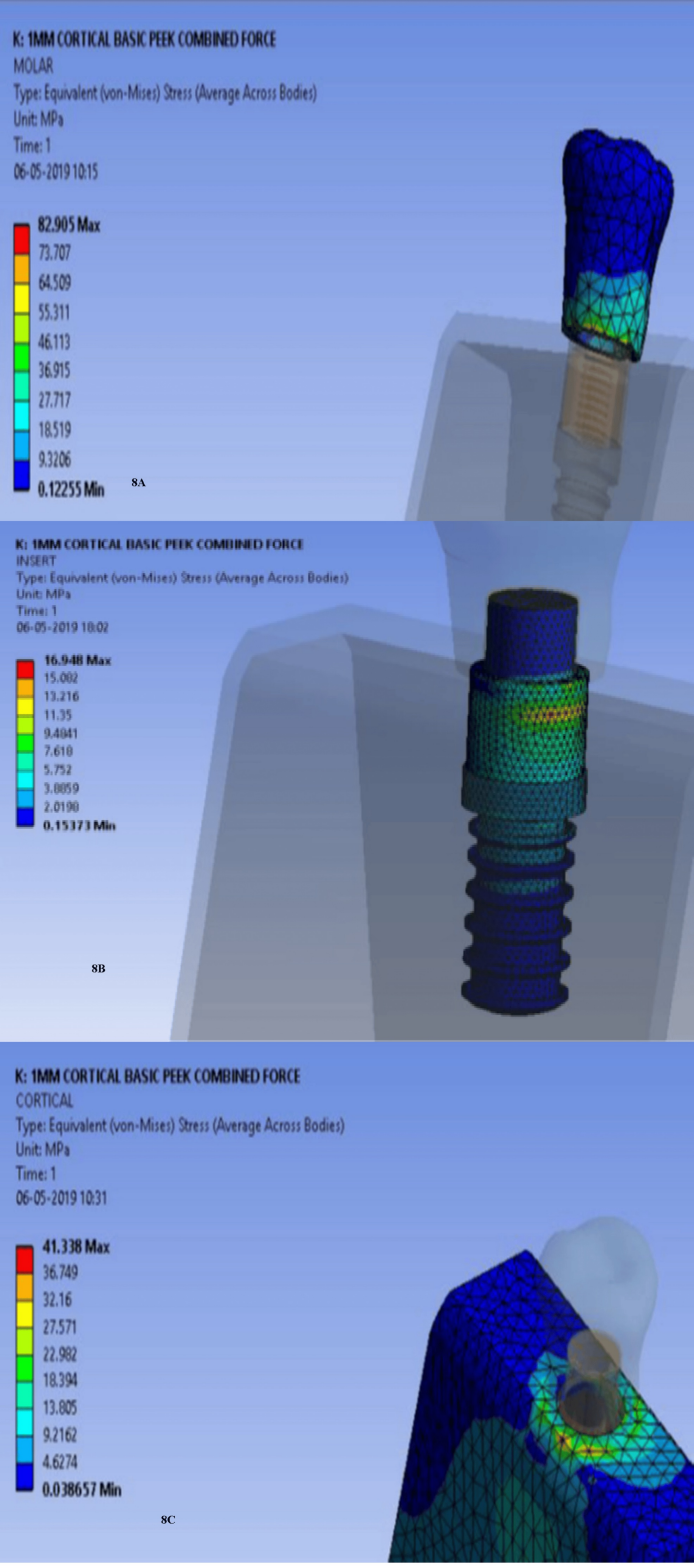
Maximum stress concentration for unmodified PEEK with low-density bone: (8A) cervical region of the prosthesis, (8B) implant-abutment interface, and (8C) surrounding bone PEEK: polyether ether ketone

**Figure 9:**
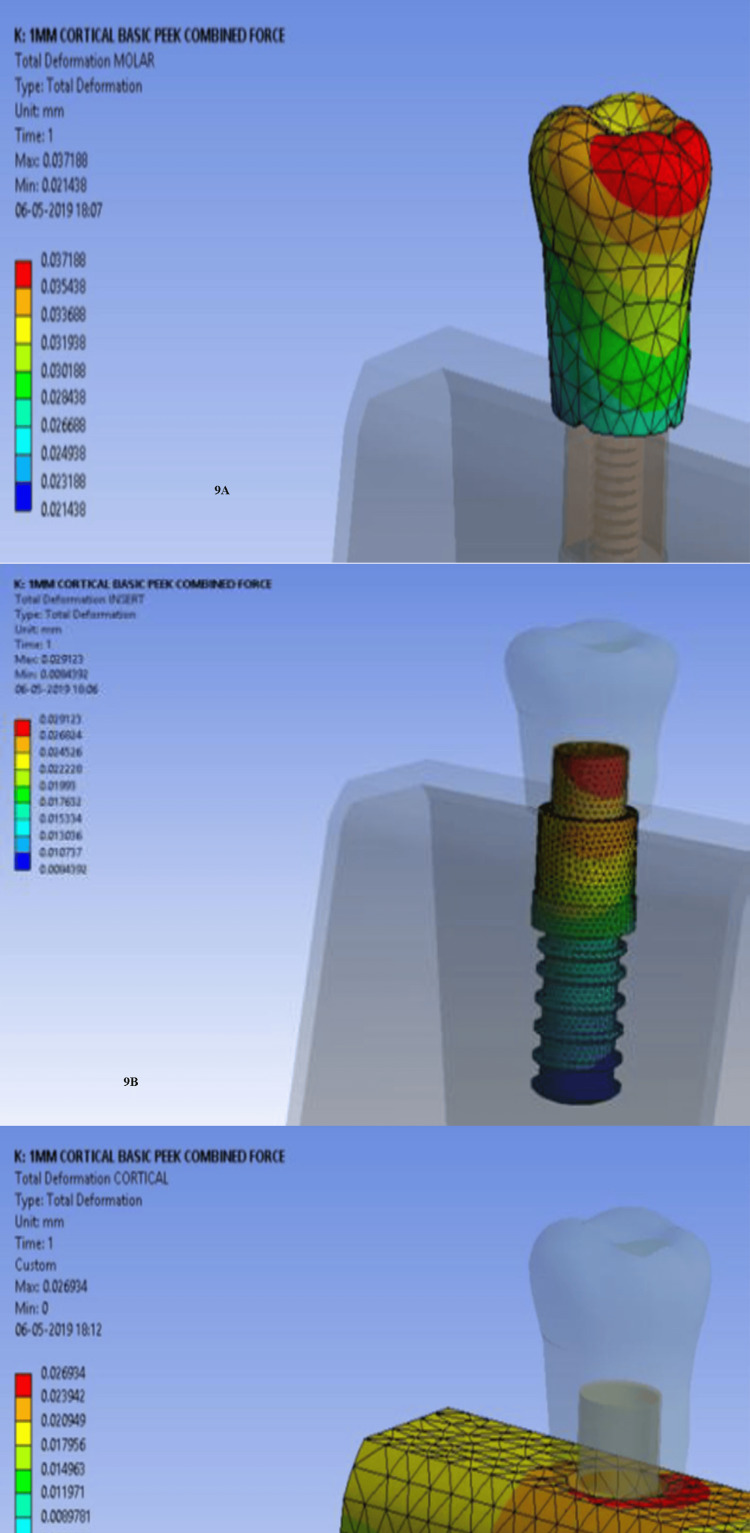
Maximum deformation with unmodified PEEK: (9A) occlusal surface of the prosthesis, (9B) implant-abutment interface, and (9C) surrounding cortical bone PEEK: polyether ether ketone

Stress was most concentrated at the cervical prosthesis region (Figure 8A), the implant-abutment interface (Figure 8B), and the implant neck region (Figure 8C), especially in low-density bone. Deformation followed a similar pattern. A similar pattern was noted for deformation. Maximum deformation occurred in the unmodified PEEK group, specifically at the occlusal surface of the prosthesis (Figure 9A), the implant-abutment interface (Figure 9B), and the surrounding cortical bone (Figure 9C).

Stress shielding occurs when the implant's stiffness (Young's modulus) differs significantly from that of the surrounding bone, leading to reduced mechanical stimulation and bone resorption. Young's modulus of unmodified PEEK (3-4 GPa) [13] is considerably lower than that of cortical bone (14 GPa) [4], contributing to potential stress shielding. In contrast, composite PEEKs, especially CFR-PEEK (12-18 GPa), offer moduli closer to bone, potentially reducing stress shielding and enhancing bone remodeling for improved osseointegration [25].

The highest stress shielding and potential for marginal bone loss were noted with unmodified PEEK, while CFR-PEEK demonstrated the most favorable biomechanical behavior, suggesting a lower risk for bone loss. These findings reject the null hypothesis and highlight significant differences in mechanical response among the materials studied.

Unmodified PEEK offers several advantages [26], including being lightweight (1.32 g/cm³), non-metallic, non-allergenic, and chemically stable, but it lacks bioactivity, limiting its bone integration potential. Composite PEEKs address this through biofunctional additives.

The findings of this study are in alignment with previous studies. Morrison et al. revealed that CFR-PEEK provides superior strength and a modulus closer to bone. Studies suggest it supports osteoblast activity without adverse effects [27]. According to Lin et al., GFR-PEEK enhances osteocalcin production, aiding bone formation [28]. An experiment by Bakar et al. found that 30% of HA possesses an elastic modulus similar to the human cortical bone [29]. The HA composite can enhance the growth of osteoblasts, according to Zhang et al. [30].

Despite the accuracy of FEA, this study has limitations. The implant was assumed to have 100% osseointegration, which is rarely the case clinically. The used materials were considered to be isotropic and homogeneous. Furthermore, the static loads that were applied differed from the dynamic loading, which better simulates functional conditions.

## Conclusions

This FEA demonstrated that implant material composition and bone density significantly influence stress distribution and deformation patterns around dental implants. Among the materials tested, CFR-PEEK exhibited the most favorable biomechanical performance, with the lowest von Mises stress and deformation under all loading conditions, followed by GFR-PEEK, Sr-HA-PEEK, and HA-PEEK. Unmodified PEEK consistently showed the highest stress concentrations and deformation, particularly in low-density bone, indicating a greater potential for stress shielding and marginal bone loss.

The findings confirm that composite PEEK implants, especially CFR-PEEK, better mimic the mechanical behavior of cortical bone, reduce stress shielding effects, and thus may promote more favorable conditions for bone remodeling and long-term implant success. Although unmodified PEEK offers advantages like low weight, biocompatibility, and chemical stability, its lack of bioactivity and mechanical mismatch with bone limit its clinical applicability.
